# ESR investigation of sucrose radicals produced by 0.25−4.5 Gy doses of X-ray irradiation

**DOI:** 10.1093/jrr/rru018

**Published:** 2014-03-26

**Authors:** Kouichi Nakagawa, Ken Kobukai, Yuzuru Sato

**Affiliations:** 1 Department of Radiological Life Sciences, Graduate School of Health Sciences, Hirosaki University, 66-1 Hon-cyo, Hirosaki, 036-8564, Japan; 2 Department of Health Sciences, Hirosaki University, 66-1 Hon-cyo, Hirosaki, 036-8564, Japan

**Keywords:** ESR, sucrose, radical, X-ray, irradiation, low dose, EPR

## Abstract

We investigated stable radicals produced by 0.25−4.5 Gy doses of X-ray irradiation of sucrose. Electron spin resonance (ESR) is able to observe the signal from sucrose irradiated at 0.25 Gy. The ESR signal intensity of the radicals is related to the accumulated dose, and it increases linearly with increasing absorbed dose. In addition, we examined the effect of dose rate (0.50−1.5 Gy/min) on the signal intensity of the irradiated sucrose. The stable radical production did not exhibit dose rate dependence. In addition, the peak corresponding to the irradiated glucose was observed to increase more with increasing absorbed dose than the peak corresponding to irradiated fructose. Therefore, the present ESR results regarding the 0.25−4.5 Gy irradiation of sucrose provide new insights into a possible sucrose ESR dosimeter.

## INTRODUCTION

The interaction between a material and radiation is an important topic in radiological science [1]. Stable radicals produced by radiation–material interactions can be reliably measured by electron spin resonance (ESR) spectroscopy. In ESR dosimetric research, the signal is associated with dose accumulation and stability. It is well known that irradiated sucrose (sugar) produces stable radicals. The signal intensity was measured after one year [1, 2]. During the year, the samples were kept in ESR tubes with caps at ambient temperature. In addition, multiradical productions have been experimentally reported by Nakagawa and co-workers [1–7].

Sucrose is a disaccharide composed of a six-membered (glucose) and a five-membered (fructose) ring. Although several radicals of irradiated sucrose have been detected after high doses of irradiation [8–9], radical identification is more complicated than in alanine because of the more complicated chemical and molecular structures. A dose <10 Gy in relation to the ESR results may provide a better understanding of the radicals produced by irradiation. The majority of the investigations have focused on relatively high doses of γ-irradiation or X-ray irradiation of sucrose; however, no study has employed ∼1 Gy dose of irradiation. Therefore, a dose <5 Gy for sucrose may provide new understanding of the ESR dosimeter.

We investigated the effects of 0.25−4.5 Gy doses of X-ray irradiation on polycrystalline sucrose using ESR to find how irradiation produces stable radicals. We further analyzed the ESR results in order to reveal the nature of the irradiation that produces stable radicals. A dose <5 Gy for sucrose will provide detailed understanding of the ESR dosimeter. In addition, we examined the ESR intensity regarding dose rate dependence of the irradiated sucrose. The ESR results are discussed in relation to the monosaccharides glucose and fructose, which are the components of sucrose.

## MATERIALS AND METHODS

### Sample preparation

Sucrose (molecular weight: 342) was purchased from Wako Pure Chemical Industries Ltd, Japan, and was used as received. Each polycrystalline sample (0.50 g) was wrapped in a plastic sheet to ensure uniform irradiation [1]. A sample quantity of 0.50 g was chosen to obtain optimum ESR signals in a cavity. The number of moles of sucrose was 1.46 × 10^−3^.

### Irradiations

Samples were irradiated using a HITACHI MBR-1520R X-ray instrument (Ibaragi, Japan) at 150 kV and 20 mA. Three different dose rates (0.50, 1.0 and 1.5 Gy/min) were used. Low-energy radiation was filtered using a 0.3-mm copper and 0.5-mm aluminum filter. Doses of 0.25, 0.50 and 1.0 Gy were delivered stepwise. All irradiated samples were kept at ambient temperature for 1 d before ESR measurements.

### ESR measurements

A sample quantity of 0.50 g in the ESR tube filled the TE_011_ cavity. After irradiation, the samples were put into standard ESR tubes (o.d. 4.7 mm, i.d. 3.6 mm, JEOL Datum Co., Japan). The radicals were measured using a JEOL RE 3X 9 GHz ESR spectrometer. The ESR measurements were performed at least 1 d after the irradiation because, according to previous reports [1–7], time dependence of the signal is negligible. The resonance frequency was measured using a microwave frequency counter of type EMC-14 (Echo Electronics Co., Ltd, Japan). All processes of irradiation and measurements were carried out at ambient temperature. The details of the ESR measurements are also described elsewhere [1–7].

## RESULTS

**Fig. 1.**
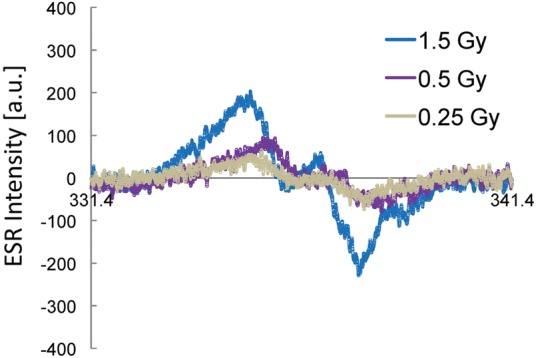
Representative ESR spectra of sucrose radicals produced by 0.25−1.5 Gy doses of X-ray irradiation. Sucrose (0.50 g) was irradiated with X-ray irradiation; the dose rate was 0.50 Gy/min. The doses are also indicated.

Figure 1 shows 0.25−1.5 Gy doses of irradiation of sucrose; the dose rate was 0.50 Gy/min. Each ESR spectrum was obtained by a single scan at ambient temperature. Although the signals are weak, ESR is able to observe signals from the irradiation of sucrose with a dose as low as 0.25 Gy. The ESR spectrum obtained at 0.25 Gy dose of irradiation shows a very weak and structureless signal. The ESR signals increase with increasing absorbed dose, as shown in Fig. 1.

**Fig. 2.**
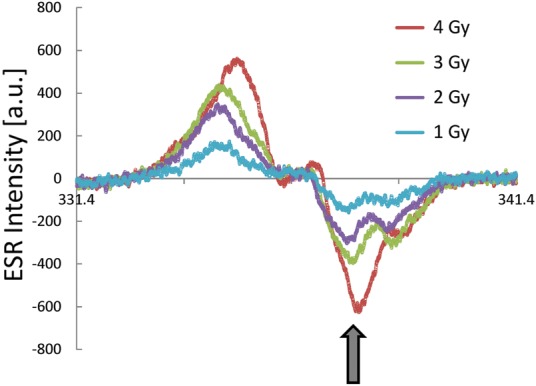
Representative ESR spectra of sucrose radicals produced by 1.0−4.0 Gy doses of X-ray irradiation. Various doses are indicated. The dose rate was 1.0 Gy/min, and the mass of each sample was 0.50 g.

Figure 2 indicates the ESR spectra obtained by 1.0−4.0 Gy doses of irradiation of sucrose; the dose rate was 1.0 Gy/min. The arrow indicates that the peak intensity is weak at 1.0 Gy dose of irradiation. Then, the arrow peak increases, and it is greater than that of the next high-field peak. The arrow signal increases with the absorbed dose when going from 1.0 to 4.0 Gy. These are very interesting phenomena of the irradiated sucrose. In addition, the ESR spectral pattern of the 4.0 Gy dose of irradiation is identical to that published in previous reports [1, 8].

**Fig. 3.**
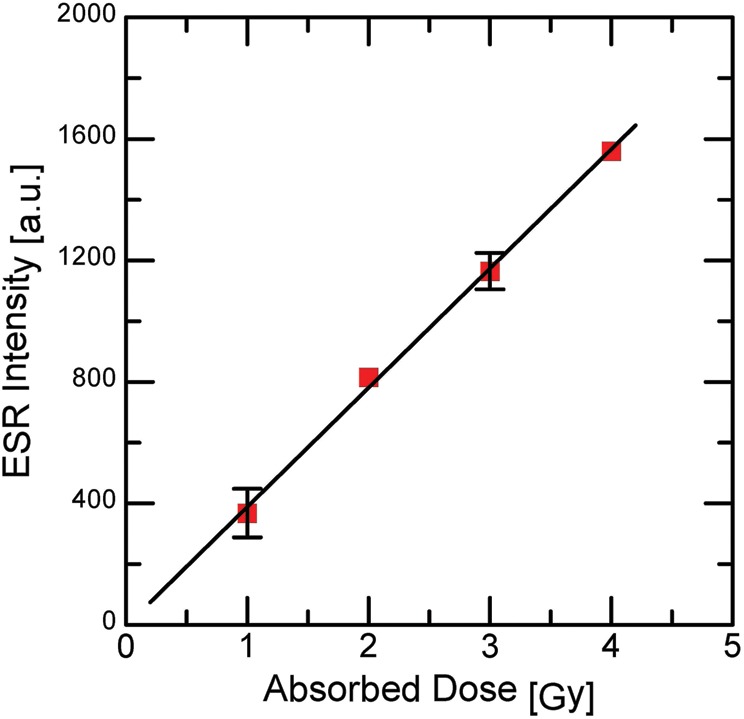
Plot of ESR intensity of the irradiated sucrose obtained by X-ray irradiation as a function of the dose; the dose rate was 1.0 Gy/min.

Figure 3 shows ESR signal intensity as a function of absorbed dose. The ESR signal intensity increases linearly with increasing absorbed dose. In addition, linear behavior was observed at 0.25 Gy dose of irradiation. The results are favorable for an ESR dosimeter at this dose. The representative error bars indicate a possible variation due to the consecutive experiments (e.g. sampling, irradiation dose of <0.50 Gy, and signal-to-noise ratio of the ESR spectrum).

**Fig. 4.**
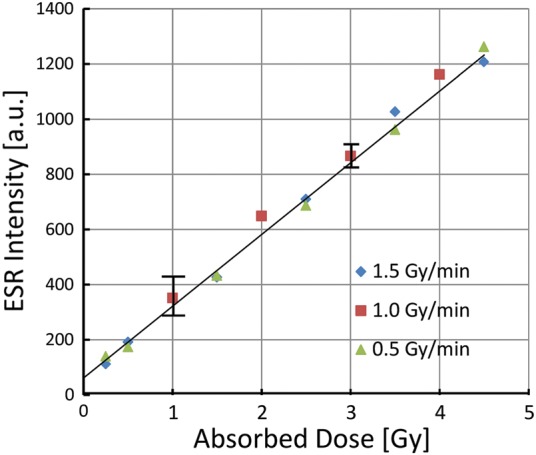
Plot of the ESR intensity as a function of dose at various dose rates. The dose rate was changed from 0.50 Gy/min to 1.5 Gy/min. The representative error bars are indicated.

Moreover, we also examined the ESR intensity regarding the dose rate dependence of the irradiated sucrose. The dose rates were changed from 0.50 to 1.5 Gy/min. The ESR spectra were obtained for 0.25−4.5 Gy doses of X-ray irradiation. The measured ESR intensity increases with increasing absorbed dose, as shown in Fig. 4. The signal intensity was found to be independent of the dose rates for stable radical production.

## DISCUSSION

We investigated the stable radicals produced by 0.25−4.5 Gy doses of irradiation of sucrose for the first time. The ESR is able to observe the signal from sucrose irradiated at 0.25 Gy. The irradiation dosage was placed at 0.25 or 0.50 Gy stepwise at a dose rate of 0.50 Gy/min or 1.0 Gy/min. The ESR signal intensity is proportional to the accumulation of the dosage. The linear increase of the ESR intensity in relation to the irradiation dosage is a good indication for an ESR dosimeter (Figs 3 and 4).

**Fig. 5.**
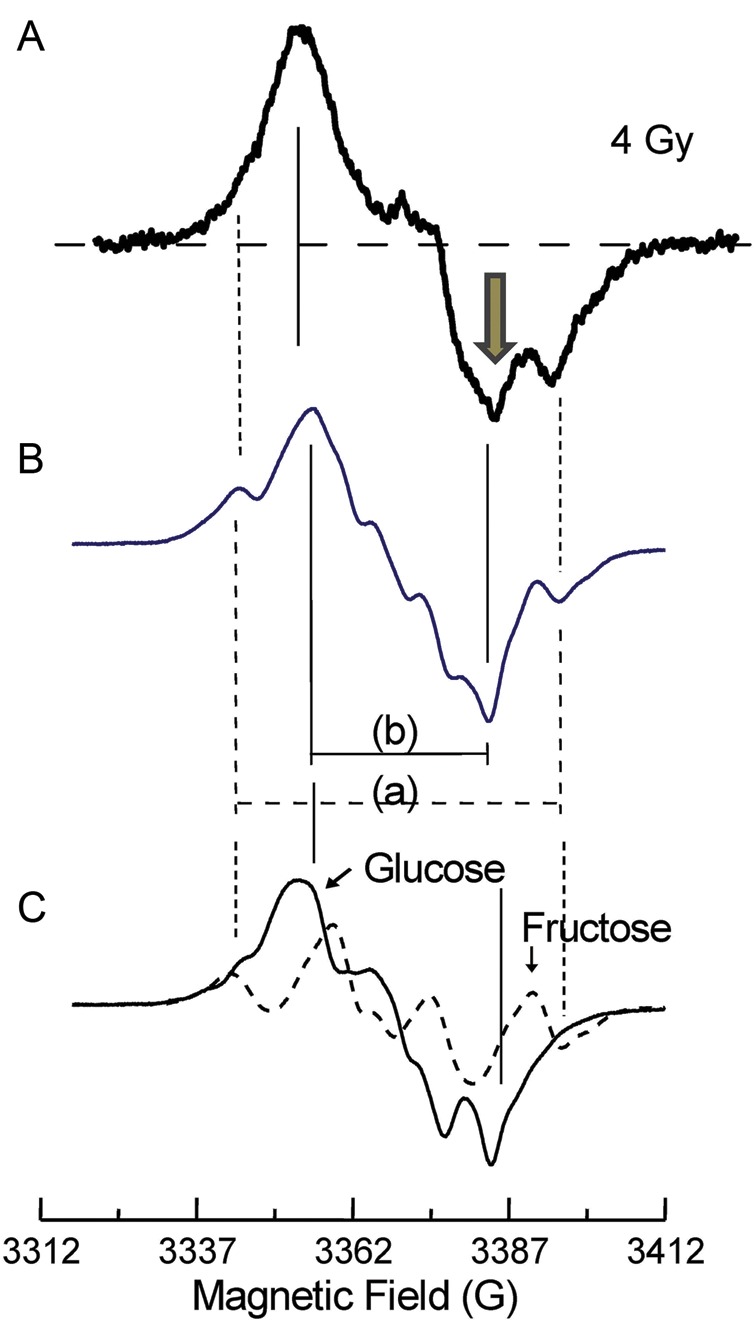
ESR spectrum (**A**) obained by X-ray irradiation (4.0 Gy, 1.0 Gy/min) of sucrose. Spectrum (**B**) is a result of spectral addition of γ-irradiated glucose and fructose shown in (**C**). Both spectra were observed with the irradiation of glucose (solid line) and fructose (dashed line). ESR spectra (B) and (C) were taken from [1]. It is noted that 10 G is equal to 1 mT.

Furthermore, the signal intensity indicated by the arrow increases more significantly than that seen with the 1.0 Gy dose of irradiation, as shown in Fig. 2. The arrow corresponds to the irradiated glucose (solid line), as shown in Fig. 5 [1]. When 0.25−4.5 Gy doses of irradiation were performed, the peaks corresponding to the irradiated glucose signal (solid line) increased more with increasing absorbed dose than those corresponding to the irradiated fructose (dashed line) (Fig. 5). Thus, the stable radicals are favorably located on the side of the six-membered ring. In addition, it is true that irradiated glucose and fructose may not be in direct relation to the irradiated sucrose. The present results of 0.25−4.5 Gy doses of irradiation of sucrose showed the detailed processes of stable radical accumulation. Therefore, the present ESR investigation of the 0.25–4.5 Gy doses of irradiation of sucrose provides new insights into the sucrose ESR dosimeter.

## FUNDING

Funding to pay the Open Access publication charges for this article was provided by a Grant-in-Aid for Exploratory Research (24650247) and for Scientific Research (B) (25282124) from the Japan Society for the Promotion of Science (JSPS) (K.N.).

## References

[1] Nakagawa K, Nishio T (2000). Electron paramagnetic resonance investigation of sucrose irradiated with heavy ions. Radiat Res.

[2] Nakagawa K, Sato Y (2005). Investigation of heavy-ion induced sucrose radicals by electron paramagnetic resonance. Radiat Res.

[3] Nakagawa K, Anzai K (2010). EPR investigation of radical-production cross sections for sucrose and L-alanine irradiated with X-rays and heavy ions. Appl Magn Reson.

[4] Karakirova Y, Nakagawa K, Yordanov ND (2010). EPR and UV spectroscopic investigations of sucrose irradiated with heavy-ion particles. Radiat Meas.

[5] Nakagawa K (2000). Effect of heavy ion irradiation on sucrose. Chem Letts.

[6] Nakagawa K, Ikota N, Anzai K (2008). Sucrose and L-alanine radical-production cross section regarding heavy-ion irradiation. Spectrochim Acta Part A.

[7] Nakagawa K, Ikota N, Sato Y (2008). Heavy-ion induced sucrose and L-α-alanine radicals investigated by electron paramagnetic resonance. Appl Magn Reson.

[8] Nakajima T, Otsuki T (1989). Dosimetry for radiation emergencies: radiation-induced free radicals in sugar of various countries and the effect of pulverizing on the ESR signal. Appl Radiat Isot.

[9] Nakajima T (1989). Possibility of retrospective dosimetry for persons accidentally exposed to ionizing radiation using electron spin resonance of sugar and mother-of-pearl. Br J Radiol.

